# RPP25 as a Prognostic-Related Biomarker That Correlates With Tumor Metabolism in Glioblastoma

**DOI:** 10.3389/fonc.2021.714904

**Published:** 2022-01-12

**Authors:** Dongdong Xiao, Jingnan Wu, Hongyang Zhao, Xiaobing Jiang, Chuansheng Nie

**Affiliations:** Department of Neurosurgery, Union Hospital, Tongji Medical College, Huazhong University of Science and Technology, Wuhan, China

**Keywords:** RPP25, glioblastoma multiforme, pan-cancer analysis, prognosis, GSEA

## Abstract

RPP25, a 25 kDa protein subunit of ribonuclease P (RNase P), is a protein-coding gene. Disorders associated with RPP25 include chromosome 15Q24 deletion syndrome and diffuse scleroderma, while systemic sclerosis can be complicated by malignancy. However, the functional role of RPP25 expression in glioblastoma multiforme (GBM) is unclear. In this study, comprehensive bioinformatics analysis was used to evaluate the impact of RPP25 on GBM occurrence and prognosis. Differential analysis of multiple databases showed that RPP25 was commonly highly expressed in multiple cancers but lowly expressed in GBM. Survival prognostic results showed that RPP25 was prognostically relevant in six tumors (CESC, GBM, LAML, LUAD, SKCM, and UVM), but high RPP25 expression was significantly associated with poor patient prognosis except for CESC. Analysis of RPP25 expression in GBM alone revealed that RPP25 was significantly downregulated in GBM compared with normal tissue. Receiver operating characteristic (ROC) combined with Kaplan-Meier (KM) analysis and Cox regression analysis showed that high RPP25 expression was a prognostic risk factor for GBM and had a predictive value for the 1-year, 2-year, and 3-year survival of GBM patients. In addition, the expression of RPP25 was correlated with the level of immune cell infiltration. The gene set enrichment analysis (GSEA) results showed that RPP25 was mainly associated with signalling pathways related to tumor progression and tumor metabolism.

## Introduction

RPP25 is an important auto-antigenic component of the Th/To complex. The Th/To antigen complex is a multi-protein RNA (RNase-MRP) complex composed of catalytic RNA and at least 10 protein components (1, 2). RNase-MRP is a widely expressed eukaryotic endo ribonuclease that specifically cleaves a variety of RNAs, including rRNA, mRNA, and mitochondrial RNA. In patients with a systemic autoimmune rheumatic disease (SARD), almost all protein components of RNase-MRP and evolutionarily related ribonuclease P (RNase P) complexes have been reported as autoantibody targets (3–5). However, studies targeting RPP25 are rarely reported.

Glioblastoma multiforme (GBM) is one of the most common, most lethal, and least prognostic subtypes of glioma (6, 7). In approximately 55% of gliomas, the median survival is only 14–16 months (8), and the 5-year survival rate is less than 5% (9). GBM is known for its highly suppressive tumor immunity, which is a critical hurdle for immunotherapy (10). A significant accumulation of suppressive regulatory T cells, M2-like tumor-associated macrophages (TAMs), and bone marrow–derived suppressor cells (MDSCs) in the tumor microenvironment has been reported to be associated with poorer overall survival in GBM patients (11). Significant reprogramming of metabolic signalling pathways is one of the most important and common features of cancer cells. Due to the rapid proliferation rate of cancer cells, there is an increased demand for energy and macromolecules. To meet these increased demands, cancer cells undergo important alterations in metabolic signalling (12). There is growing evidence that metabolic dysregulation plays an important role in the growth, proliferation, angiogenesis, and invasion of cancer cells (13–15). A series of metabolism-related risk genes could come to assess the prognosis of GBM patients in the future and could be closely related independent predictors of prognosis in GBM patients (16).

Whether RPP25 has some predictive value in tumor progression and prognosis, what is the mechanism of this, and whether RPP25 can be a prognostic predictor or therapeutic target for GBM are a series of studies that have not been reported. In recent years, the rise of cancer driver genes and signalling pathway identification based on high-throughput omics data has provided a new perspective for cancer research.

In this study, we first analysed the expression and survival prognosis of RPP25 in multiple cancers by pan-cancer analysis, and then in GBM alone, and found that RPP25 was a prognostic predictor of GBM. Gene pooling enrichment analysis revealed that RPP25 is involved in regulating tumor metabolism and tumor immune-related signalling pathways.

## Methods

### Data Collection

**Figure 1:**
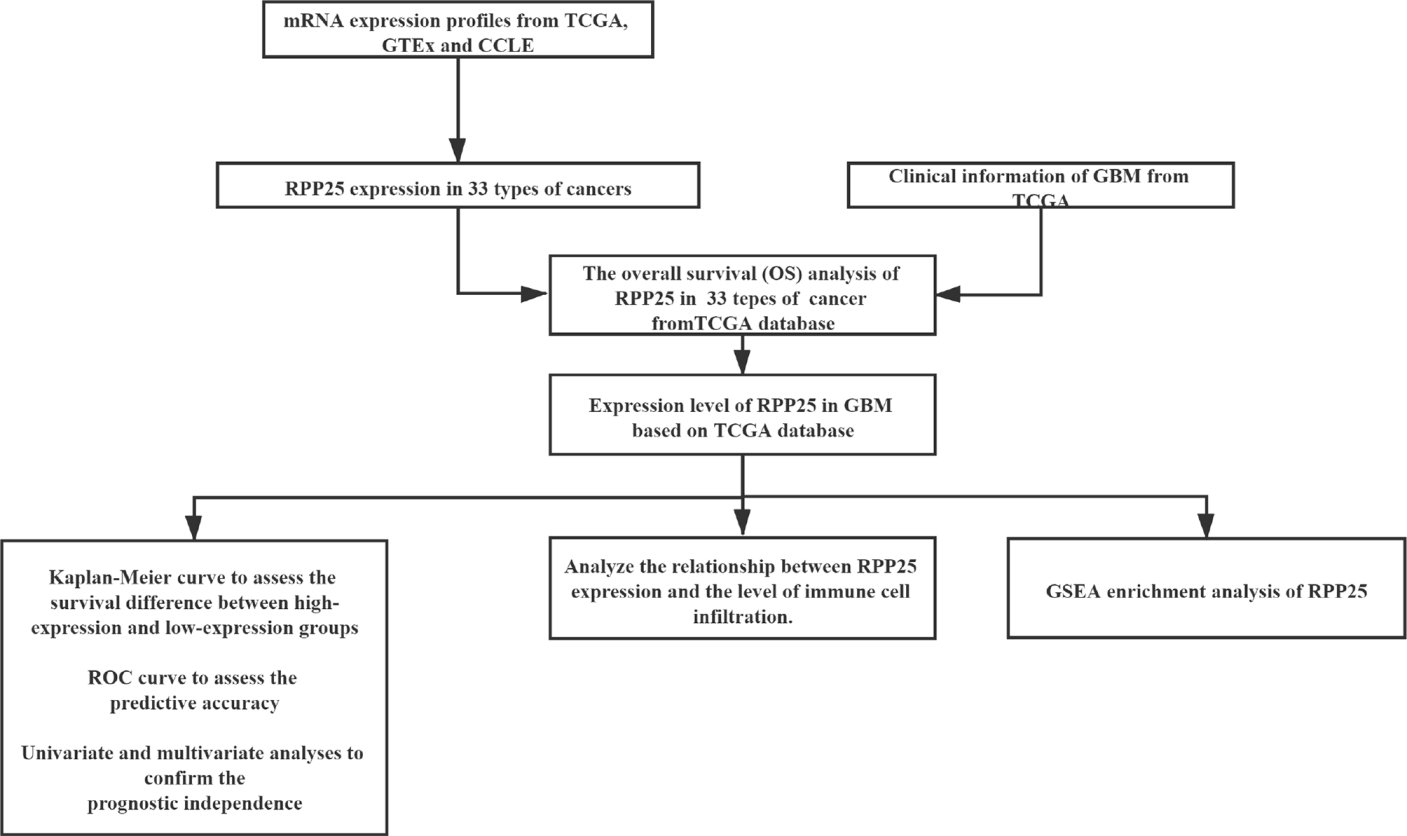
Flow diagram of this study.

All gene expression datasets were obtained from the combined databases of The Cancer Genome Atlas (TCGA) (https://portal.gdc.cancer.gov/) and Genotype-Tissue Expression (GTEx) (https://gtexportal.org/). The mRNA expression profiles of tumor tissue samples and normal tissue samples of 33 types of cancer were downloaded from TCGA and GTEx, respectively. The expression data of each tumor cell line were downloaded from the Cancer Cell Line Encyclopedia (CCLE) database (https://portals.broadinstitute.org/). All analytical methods were performed using R software v4.0.3 (R Foundation for Statistical Computing, 2020), rank sum test was used to evaluate the difference of mRNA level between the two groups and *p* < 0.05 was considered to be statistically significant. The study flowchart is illustrated in **Figure 1**.

### Kaplan-Meier Analysis

Raw counts of RNA sequencing data (level 3) and corresponding clinical information for 153 tumor samples in GBM were obtained from the TCGA dataset (https://portal.gdc.com). The log-rank test was used to examine Kaplan-Meier (KM) survival analysis comparing survival differences between two or more of the groups under investigation, and analysis with the timeROC R package was performed to compare the predictive accuracy and risk scores of the RPP25 gene. The clinical and pathological characteristics of GBM patients were displayed in **Supplementary Table 1**.

For KM curves, *p* values and hazard ratios (HRs) with 95% confidence intervals (CIs) were derived by log-rank test and univariate Cox proportional hazards regression.

### Cox Model

Univariate and multivariate Cox regression analyses and forest plots were performed using the forestplot R package to show the *p* value, HR, and 95% CI for each variable. Based on the results of the multivariate Cox proportional risk analysis, column line plots were created using the rms R package to predict the 1-year, 2-year, and 3-year total recurrence rates. The column line graphs provide a graphical representation of these factors, and the prognostic risk of individual patients can be calculated by the points associated with each risk factor.

### Tumor IMmune Evaluation Resource (TIMER) Analysis

The Tumor IMmune Estimation Resource (TIMER) database (http://timer.comp-genomics.org/) is an integrated database for the analysis of immune infiltration in different tumor types. The correlation between RPP25 expression in GBM and the level of immune cell infiltration was analysed according to the expression of biomarker genes in the tumor.

### Gene Set Enrichment Analysis (GSEA)

To observe the effect of gene expression on tumors, samples were divided into two groups of high and low expression according to gene expression, and the enrichment of Kyoto Encyclopedia of Genes and Genomes (KEGG) and hallmark pathways in the high and low expression groups were analysed using gene set enrichment analysis (GSEA).

## Results

### Expression of RPP25 in Tumors

**Figure 2:**
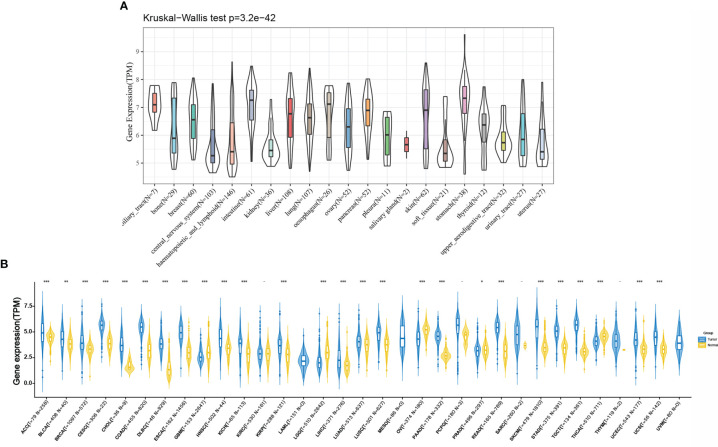
Expression of RPP25 in tumors; **(A)** Expression level of RPP25 in 21 tumors of CCLE origin; **(B)** Expression level of RPP25 in 33 tumors of TCGA+GTEx origin; *P < 0.05, **P < 0.01, ***P < 0.001, -P > 0.05.

As shown in **Figure 2A**, we retrieved the expression data of RPP25 in 21 tumor tissue cell lines from the CCLE database and analysed the expression levels of RPP25 in the 21 tissue cell lines according to their tissue sources. Due to the small number of normal samples in TCGA, we integrated normal tissue data from the GTEx database with TCGA tumor tissue data to analyze the expression differences of RPP25 in 33 types of tumors (Sample size of each type of cancer were displayed in the x-coordinate of **Figure 2B**), and from the results (**Figure 2B**) we found that RPP25 expression was upregulated in the vast majority of tumor tissues compared to normal tissues, except for GBM, LGG, OV, and PRAD.

### Analysis of Overall Survival for RPP25 Expression in Tumors

**Figure 3:**
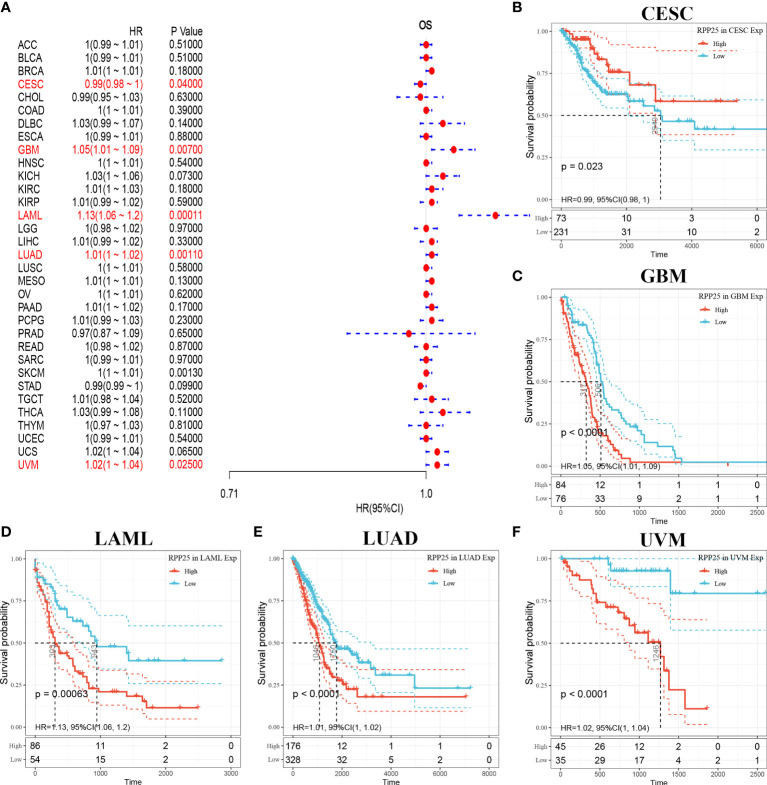
Analysis of overall survival of RPP25 in tumors; **(A)** Correlation between RPP25 expression and OS by univariate COX analysis in multiple tumors; **(B)** Kaplan-Meier curves and univariate cox regression of OS of RPP25 in CESC patients stratified by RRP25 expression; **(C)** Kaplan-Meier curves and univariate cox regression of OS of RPP25 in GBM patients stratified by RRP25 expression; **(D)** Kaplan-Meier curves and univariate cox regression of OS of RPP25 in LAML patients stratified by RRP25 expression; **(E)** Kaplan-Meier curves and univariate cox regression of OS of RPP25 in LUAD patients stratified by RRP25 expression; **(F)** Kaplan-Meier curves and univariate cox regression of OS of RPP25 in UVM patients stratified by RRP25 expression.

The relationship between RPP25 expression in 33 tumors and overall survival was first analysed using univariate survival analysis. The results of the forest plot in **Figure 3A** showed that RPP25 expression significantly affected CESC (HR = 0.99, *p* = 0.040), GBM (HR = 1.05, *p* = 0.007), LAML (HR = 1.13, *p* = 0.0001), LUAD (HR = 1.01, *p* = 0.0010), SKCM (HR = 1, *p* = 0.0013), and UVM (HR = 1.02, *p* = 0.0250) patients for overall survival. Tumor KM curves of significant correlation between RPP25 expression and patient prognosis are shown in **Figures 3B–F**. The results showed that high RPP25 expression in GBM, LAML, LUAD, SKCM, and UVM was significantly associated with poor patient prognosis, suggesting that RPP25 may be a potential pan-cancer prognostic indicator molecule.

### Expression of RPP25 in Glioblastoma Multiforme (GBM)

**Figure 4:**
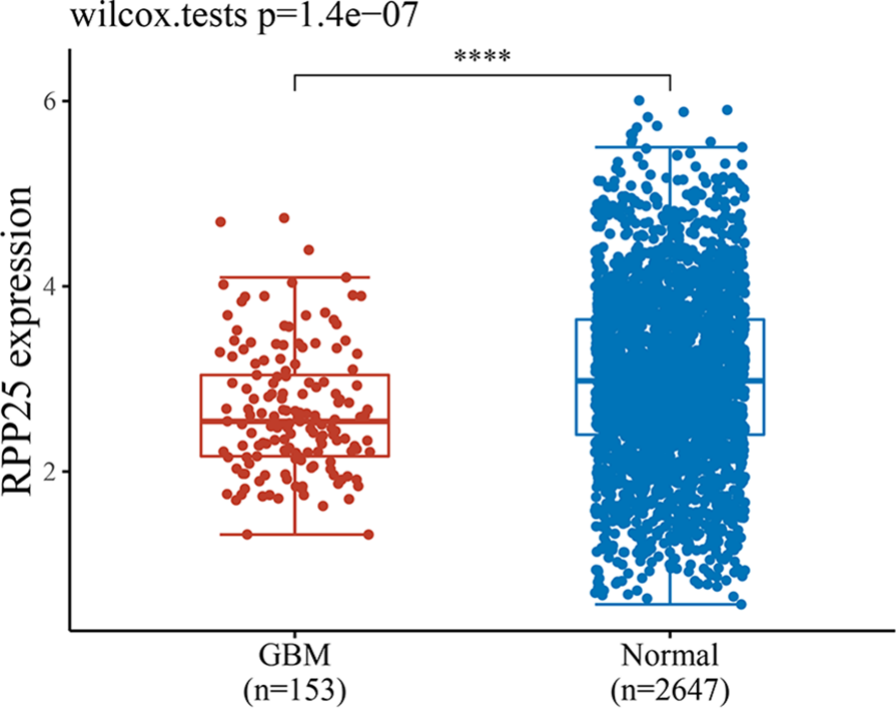
Expression level of RPP25 in GBM; ****P < 0.001.

GBM-related data from the TCGA and GTEx databases were downloaded, and a total of 153 tumor samples and 2647 normal samples were obtained. A rank sum test statistical analysis was performed using R software v4.0.3, and the results showed that RPP25 was lowly expressed in GBM cancer tissues compared to normal tissues (**Figure 4**).

### Effect of RPP25 Expression on GBM Survival Prognosis

**Figure 5:**
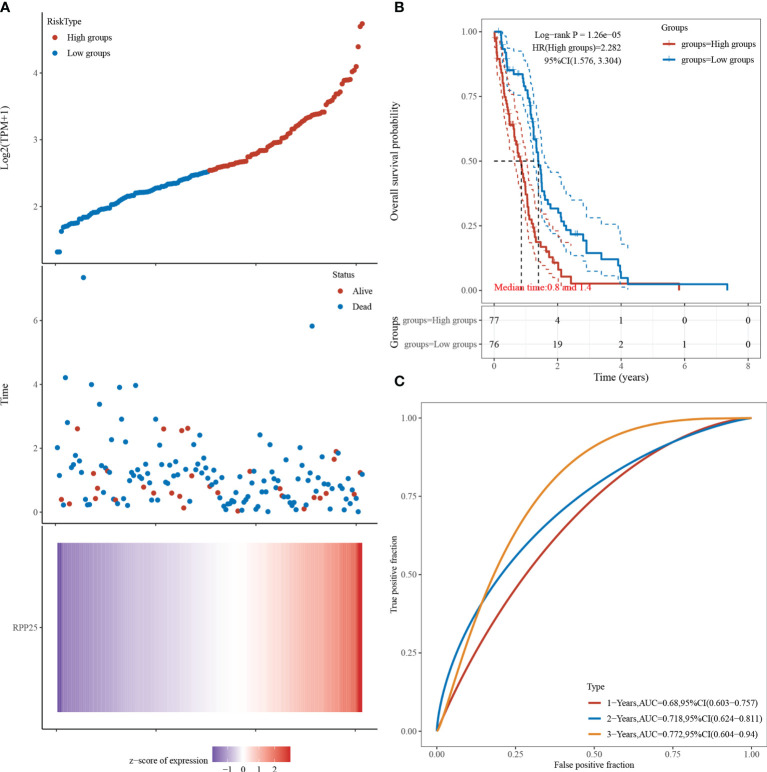
RPP25 expression and GBM survival analysis; **(A)** RPP25 expression versus survival time and survival status in GBM patients from TCGA datasets; **(B)** KM survival curve distribution of RPP25 expression in TCGA dataset visualized by R packages survival and survminer; **(C)** ROC curve and AUC of RPP25 at different times.

**Figure 5A** visualizes the RPP25 gene with survival time and survival status using the ggrisk R package. When RPP25 expression is sorted from low to high, the corresponding middle scatter plot from left to right presents a trend of patients dying more with shorter time. The results in **Figure 5B** suggest that the higher the RPP25 expression, the worse the prognosis, and hence the gene can be identified as a risk factor. The receiver operating characteristic (ROC) curves suggest that the area under curve (AUC) values for 1-year, 2-year, and 3-year survival are 0.680, 0.718, and 0.772 **Figure 5C**, respectively, indicating that the model has good accuracy.

### A Prognostic Risk Model for GBM

**Figure 6:**
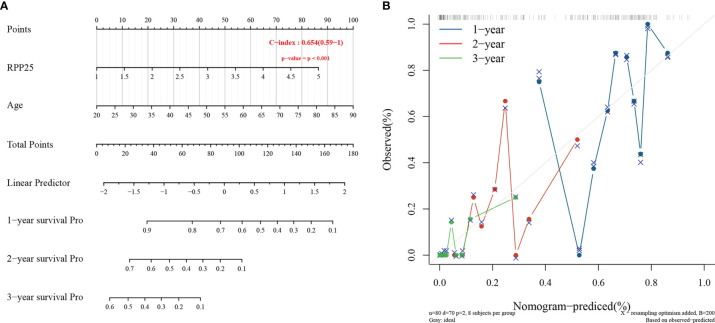
RPP25 risk prediction column line chart and prediction model; **(A)** column line chart showing risk factors affecting the prognosis of GBM patients; **(B)** column line chart model calibration curve.

The Cox proportional risk model was used to conduct single-factor and multi-factor survival analyses, respectively, and RPP25 and age were identified as GBM prognostic risk factors (**Table 1**), and RPP25 was identified as a high-risk factor. Then, a prediction model and calibration curve for 1-year, 2-year, and 3-year survival probability were established (**Figure 6**) with diagonal dotted lines representing ideal programs and blue, red and orange lines representing observed 1y, 2y and 3y nomogram. The results showed that the nomogram based on the age and RPP25 expression had a favorable ability to predict prognosis for GBM patients of 1y, 2y and 3y survival.

**Table 1:** Correlation of RPP25 expression and prognosis in GBM with diverse clinicopathological factors by Kaplan-Meier plotter.

Parameter	Univariate analysis			Multivariate analysis		
	HR	95% CI	P	HR	95% CI	P
RPP25	1.4775	1.1481-1.9016	0.0024	1.6673	1.1969-2.3225	0.0025
Age	1.0249	1.0103-1.0397	0.0007	1.0338	1.0109-1.0573	0.0036
Gender	0.9286	0.6372-1.3534	0.7000	1.1152	0.6398-1.9437	0.7005
Race	1.0066	0.6442-1.5728	0.9769	1.0422	0.5348-2.0309	0.9034
New Tumor	0.7708	0.4197-1.4156	0.4013	0.7638	0.4141-1.40904	0.3885

### Relationship Between RPP25 Expression and the Level of Immune Cell Infiltration

**Figure 7:**
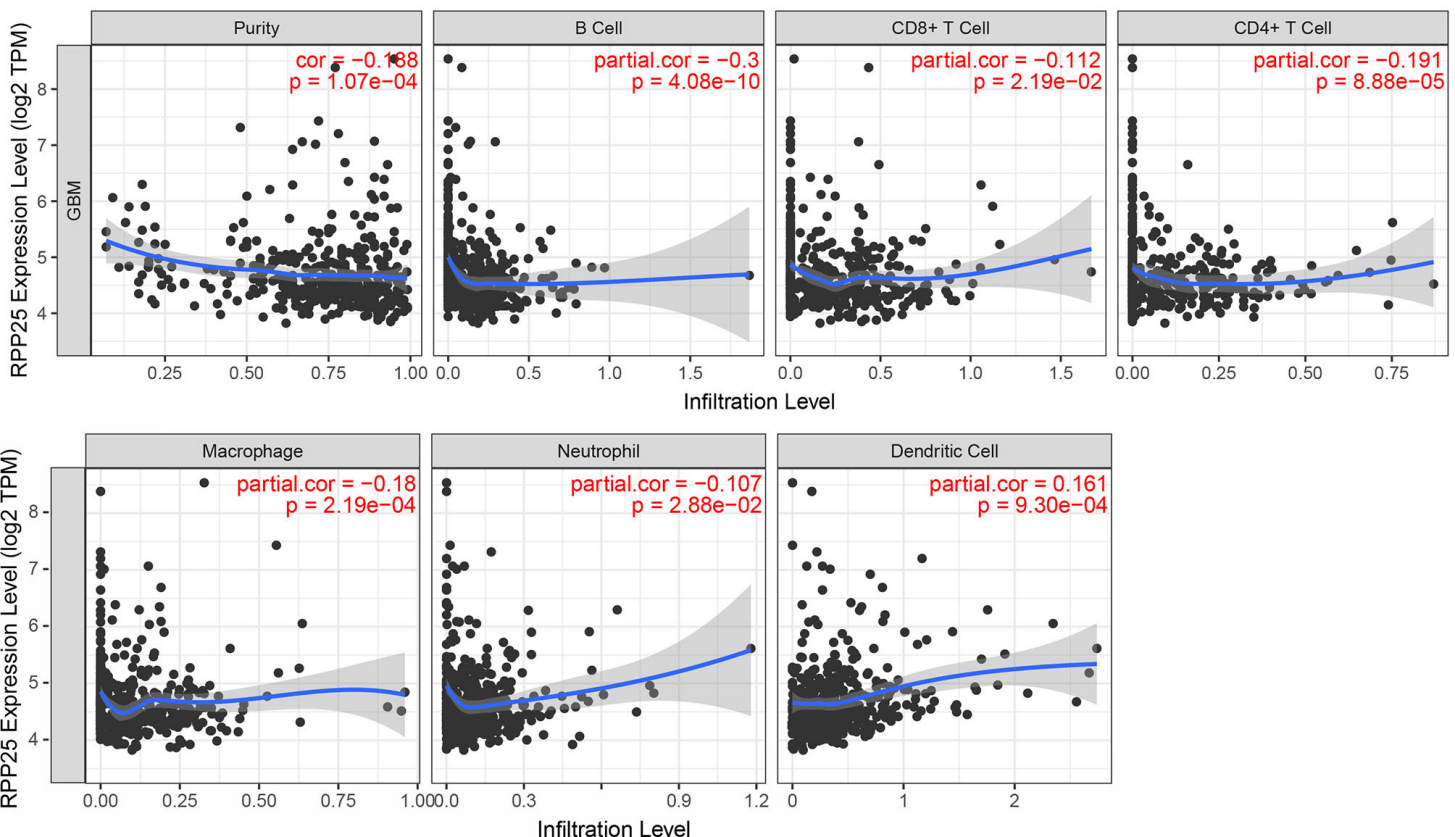
Relationship between RPP25 expression and the level of immune cell infiltration.

TIMER was used to study the correlation between RPP25 expression and the level of immune cell infiltration in GBM. Overall, RPP25 expression was significantly correlated with the level of immune cell infiltration (**Figure 7**). Specifically, RPP25 expression was weakly positively correlated with dendritic cell infiltration (*R=*0.161, *p*= 9.30×10^-4^) and weakly negatively correlated with B cell (*R=*-0.300, *p*= 4.08×10^-10^), while negatively correlated with CD4+ T cell (*R=*-0.191, *p*= 8.88×10^-5^), macrophage (*R=*-0.18, *p*= 2.19×10^-4^), CD8+ T cell (*R=*-0.112, *p*= 2.19×10^-2^) and neutrophil (*R=*-0.107, *p*= 2.88×10^-2^). These results suggested that RPP25 was correlated with immune cell infiltration in GBM.

### Gene Set Enrichment Analysis (GSEA)

**Figure 8:**
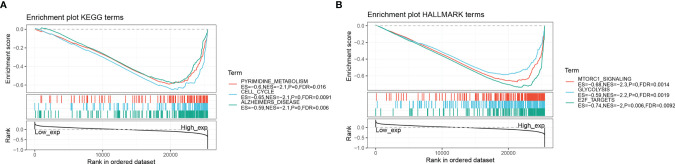
Gene set enrichment analysis. **(A)** KEGG; **(B)** Hallmark.

**Tables 2** and **3** show the top 10 most abundant signalling pathways or biological processes, respectively, ranked according to normalized enrichment score (NES) values. As shown in **Figure 8A**, the three KEGG signalling pathways most significantly associated with high RPP25 expression are given. Among them, high RPP25 expression was enriched in pyrimidine metabolism, cell cycle, and Alzheimer’s disease–related pathways. The three hallmark pathways most significantly associated with high RPP25 expression are shown in **Figure 8B**. Among them, high RPP25 expression was also positive for the mechanistic target of rapamycin (mTOR) complex 1 (mTORC1) signalling system, glycolytic pathway, and E2F targets.

**Table 2:** The information of KEGG terms from top 10 GSEA enrichment analysis.

Term	ES	NES	NP	FDR	FWER
KEGG_PYRIMIDINE_METABOLISM	-0.5968	-2.1275	0	0.0165	0.0120
KEGG_CELL_CYCLE	-0.6547	-2.1189	0	0.0091	0.0130
KEGG_ALZHEIMERS_DISEASE	-0.5882	-2.1085	0	0.0060	0.0130
KEGG_PROTEASOME	-0.8075	-2.0930	0	0.0050	0.0150
KEGG_HUNTINGTONS_DISEASE	-0.5879	-2.0793	0	0.0048	0.0160
KEGG_DNA_REPLICATION	-0.8255	-2.0395	0.0020	0.0092	0.0330
KEGG_P53_SIGNALING_PATHWAY	-0.5436	-2.0312	0	0.0092	0.0360
KEGG_AMYOTROPHIC_LATERAL_SCLEROSIS_ALS	-0.5453	-2.0110	0	0.0114	0.0460
KEGG_OXIDATIVE_PHOSPHORYLATION	-0.6771	-1.9971	0.0059	0.0124	0.0540
KEGG_PARKINSONS_DISEASE	-0.6664	-1.9954	0.0059	0.0113	0.0550

**Table 3:** The information of HALLMARK terms from top 10 GSEA enrichment analysis.

Term	ES	NES	NP	FDR	FWER
HALLMARK_MTORC1_SIGNALING	-0.6774	-2.2613	0	0.0014	0.0010
HALLMARK_GLYCOLYSIS	-0.5906	-2.1675	0	0.0019	0.0030
HALLMARK_E2F_TARGETS	-0.7409	-2.0366	0	0.0092	0.0170
HALLMARK_MYC_TARGETS_V2	-0.7800	-2.0322	0	0.0073	0.0180
HALLMARK_MYC_TARGETS_V1	-0.7264	-2.0175	0	0.0070	0.0210
HALLMARK_OXIDATIVE_PHOSPHORYLATION	-0.6711	-2.0012	0.0040	0.0088	0.0280
HALLMARK_DNA_REPAIR	-0.5826	-1.9959	0	0.0082	0.0310
HALLMARK_UNFOLDED_PROTEIN_RESPONSE	-0.5939	-1.9677	0	0.0105	0.0410
HALLMARK_G2M_CHECKPOINT	-0.6757	-1.9288	0	0.0136	0.0570
HALLMARK_REACTIVE_OXYGEN_SPECIES_PATHWAY	-0.6200	-1.9265	0	0.0124	0.0580

## Discussion

Cellular metabolism is capable of meeting the demands of tissue homeostasis and growth. One of the hallmarks of cancers is their ability to reprogram cellular metabolism to acquire metabolic adaptations in response to extrinsic and intrinsic cellular information, thus providing selective growth advantage and sustained proliferative capacity (17, 18). Aerobic glycolysis is one of the most well-studied examples of a metabolic pathway reprogrammed in cancer cells, and Otto Warburg described how the cellular metabolism of cancer cells shifts from oxidation to glycolysis even in the presence of physiological levels of oxygen, termed the Warburg effect (19). This is a metabolic property of oncogenes under autonomous control in many proliferating cancer cells and tumors (20–22). If cancer cells develop a fixed dependence on the Warburg effect while non-malignant cells adapt to its inhibition, there could be a possible therapeutic direction that takes advantage of this. Moreover, according to Warburg’s basic study, cancer cells obtain energy mainly through the glycolytic pathway rather than the oxidative phosphorylation pathway, so abnormal glycolytic metabolism is one of the basic features of malignant cells (23). In addition, growth factor signalling and nutrition are effectively concentrated in mTORC1, which can regulate key processes such as cellular and organismal glycolytic metabolism, protein metabolism, lipid metabolism, and cellular autophagy (24–26). In other words, mutations in key proteins of the mTORC1 signalling pathway will lead to dysregulation of mTORC1 activity, which in turn will lead to the disruption of cellular metabolism or cell proliferation processes and further lead to the development of many metabolism-related diseases. Therefore, the mechanism of the mTORC1 signalling pathway in cell metabolism is worthy of further investigation.

In this study, we found that RPP25 can be involved in a variety of metabolic pathways and biosyntheses, including glycolysis, mTORC1 signalling, pyrimidine metabolism, proteases, and oxidative phosphorylation, by GSEA enrichment analysis of RPP25. This suggests that high RPP25 expression is involved in the positive regulation of these signalling pathways and may play a role in promoting aerobic glycolysis and affecting tumor cell metabolism by driving the upregulation of these metabolic and signalling pathways. Civita et al. (27) revealed a heterogeneous landscape of GBM by laser capture microdissection and RNA sequencing analysis, showing metabolic pathway dysregulation, which provides direct evidence that RPP25 expression in GBM may be influenced by metabolic alterations regulated through the glycolytic pathway. In addition, it was found that mTOR signal regulation plays a key role in regulating immune response, such as T cell and myeloid cell differentiation and multiple metabolic functions (28). Selective inhibition of mTOR has profound effects on immune cell populations, including CD8 + T cells, CD4 + T cells, CD3 + T cells and B cells, as well as antitumor immunity (29). It can be seen that immune recognition helps to inhibit tumor and enhance cell infiltration which acts as a molecular signal for the activation of tumor immune microenvironment (30). Combined with the results of which RPP25 expression was significantly correlated with the level of immune cell infiltration. Despite the weakly correlations, but it is still possible that RPP25 might mediate mTOR signal pathway to regulate immune response.

The above results demonstrate that RPP25 may influence tumor progression by regulating cellular metabolism, so are there more possible mechanisms for the presence of RPP25 expression in GBM? To be mentioned here is the mTORC1 signalling pathway. Abnormal developmental features of the brain, including macrocephaly (31), focal cortical dysplasia (32), and GBM (33), have been shown to be associated with mTOR signalling pathways. These defects are mainly associated with mTOR-associated cells malfunctioning upstream or downstream of the signalling cascade (33). Specifically, in the brain, mTOR is involved in the regulation of neuronal synaptogenesis, corticogenesis, and related functions (34). Many neuropsychiatric diseases and neurodevelopmental disorders, such as Alzheimer’s disease and autism (34, 35), are also associated with mTOR. In particular, alterations in mTORC1 activity caused by tuberous sclerosis mutations are associated with Alzheimer’s disease, and since RPP25 was previously mentioned as a systemic sclerosis-related gene and we also found through KEGG functional enrichment analysis that RPP25 is mainly enriched in Alzheimer’s disease–related pathways, we can also speculate that RPP25 is in GBM perhaps by affecting mTOR signalling pathways in GBM.

We also found that based on differential expression analysis, RPP25 was lowly expressed in GBM compared to normal tissues, but the results of survival analysis by KM showed that high expression of RPP25 was significantly correlated with poor prognosis of patients. The appearance of this differential may indicate that RPP25 may act at different times of tumor progression and may be a cancer suppressor in the early stage and a cancer promoter in the late stage. Some genes may be beneficial to the body, but once a tumor forms, the tumor cells may hijack the gene to protect the tumor cells. It has been suggested that cancer cells can organize telomere shortening by hijacking DNA repair pathways, thus allowing tumor cells to spread (36). Similarly, we found that RPP25 is also enriched in the DNA repair pathway, which, by association, provides evidence for this difference between the low expression of RPP25 in GBM and its poor prognosis with high expression. Due to such differences, it provides many potential drug targets for the treatment of GBM.

In some references found that overexpression of RPP25 could block cells in the G0/G1 phase to suppress cancer cell proliferation. RPP25 interacted with the P3 domain of the RNase MRP RNA and are also associated with human RNase P (37). A role for RNase MRP in yeast cell cycle regulation was reported by Schmitt and collaborators, who showed that RNase MRP plays a role in the degradation of the mRNA encoding the mitosis specific cyclin Clb2 (38).

In summary, our study mainly found that RPP25 can be a biomarker for prognosis prediction of GBM and has the potential to provide ideas for therapeutic targets in GBM. The significance of our work is to prospectively reveal which relevant signalling pathways may be associated with the mechanism of action of RPP25 in GBM, providing a bioinformatic basis for further understanding the role of RPP25 in tumor metabolism. The work is also significant in that it prospectively reveals which signalling pathways may be associated with RPP25 expression in GBM and provides a bioinformatics basis for further understanding of the role of RPP25 in tumor metabolism.

## Data Availability Statement

The original contributions presented in the study are included in the article/**Supplementary Material**. Further inquiries can be directed to the corresponding authors.

## Author Contributions

All authors listed have made a substantial, direct, and intellectual contribution to the work, and approved it for publication.

## Funding

This study has received funding from National Natural Science Foundation of China (81974390). The funders had no role in study design, data collection and analysis, decision to publish, or preparation of the manuscript.

## Conflict of Interest

The authors declare that the research was conducted in the absence of any commercial or financial relationships that could be construed as a potential conflict of interest.

## Publisher’s Note

All claims expressed in this article are solely those of the authors and do not necessarily represent those of their affiliated organizations, or those of the publisher, the editors and the reviewers. Any product that may be evaluated in this article, or claim that may be made by its manufacturer, is not guaranteed or endorsed by the publisher.
