# Supplementary material for: RPP25 as a Prognostic-Related Biomarker That Correlates With Tumor Metabolism in Glioblastoma
Source: Front Oncol. 2022 Jan 12;11:714904. doi: 10.3389/fonc.2021.714904 (PMC8790702; doi:10.3389/fonc.2021.714904)
Supplement: Supplementary file 1 [file Table_1.docx]

Supplementary Table 1

| Characteristics | | GBM（n=153） |
| --- | --- | --- |
| Status | Alive | 31 |
|  | Dead | 122 |
| Age | Mean(SD) | 59.7 (13.6) |
|  | Median[Min,Max] | 60[21,89] |
| Gender | Female | 54 |
|  | Male | 99 |
| Race | Asian | 5 |
|  | Black | 10 |
|  | White | 137 |
| New tumor event type | Progression | 64 |
|  | Recurrence | 16 |
| Radiation therapy | Radiation | 1 |
| History of neoadjuvant treatment | No neoadjuvant | 153 |
| Therapy type | Chemotherapy | 59 |
|  | Chemotherapy:Hormone Therapy | 15 |
|  | Chemotherapy:Hormone Therapy:Targeted Molecular therapy | 1 |
|  | Chemotherapy:Immunotherapy | 1 |
|  | Chemotherapy:Immunotherapy:Targeted Molecular therapy | 2 |
|  | Chemotherapy:Targeted Molecular therapy | 27 |
|  | Hormone Therapy | 6 |
|  | Hormone Therapy:Targeted Molecular therapy | 1 |
